# Elucidation of the evolutionary history of *Stipa* in China using comparative transcriptomic analysis

**DOI:** 10.3389/fpls.2023.1275018

**Published:** 2023-11-28

**Authors:** Na Sha, Zhiyong Li, Qiang Sun, Ying Han, Li Tian, Yantao Wu, Xing Li, Yabo Shi, Jinghui Zhang, Jiangtao Peng, Lixin Wang, Zhenhua Dang, Cunzhu Liang

**Affiliations:** ^1^ Key Laboratory of Ecology and Resource Use of the Mongolian Plateau, Ministry of Education of China, Collaborative Innovation Center for Grassland Ecological Security, School of Ecology and Environment, Inner Mongolia University, Hohhot, Inner Mongolia, China; ^2^ Institute of Landscape and Environment, Inner Mongolia Academy of Forestry Science, Hohhot, Inner Mongolia, China; ^3^ School of Resources and Environment, Baotou Teachers’ College, Baotou, Inner Mongolia, China

**Keywords:** *Stipa*, phylogenetic, comparative transcriptomics, evolutionary history, speciation, Qinghai-Tibet plateau, Mongolian plateau, Pleistocene

## Abstract

Phylogenetic analysis provides crucial insights into the evolutionary relationships and diversification patterns within specific taxonomic groups. In this study, we aimed to identify the phylogenetic relationships and explore the evolutionary history of *Stipa* using transcriptomic data. Samples of 12 *Stipa* species were collected from the Qinghai-Tibet Plateau and Mongolian Plateau, where they are widely distributed, and transcriptome sequencing was performed using their fresh spikelet tissues. Using bidirectional best BLAST analysis, we identified two sets of one-to-one orthologous genes shared between *Brachypodium distachyon* and the 12 *Stipa* species (9397 and 2300 sequences, respectively), as well as 62 single-copy orthologous genes. Concatenation methods were used to construct a robust phylogenetic tree for *Stipa*, and molecular dating was used to estimate divergence times. Our results indicated that *Stipa* originated during the Pliocene. In approximately 0.8 million years, it diverged into two major clades each consisting of native species from the Mongolian Plateau and the Qinghai-Tibet Plateau, respectively. The evolution of *Stipa* was closely associated with the development of northern grassland landscapes. Important external factors such as global cooling during the Pleistocene, changes in monsoonal circulation, and tectonic movements contributed to the diversification of *Stipa*. This study provided a highly supported phylogenetic framework for understanding the evolution of the *Stipa* genus in China and insights into its diversification patterns.

## 1 Introduction

The *Stipa* genus consists of temperate, herbaceous plants. This genus originated from the Pooideae subfamily and has evolved into the dominant and key species of needlegrass in the Eurasian grasslands (Lu and Wu, 1996; Nobis et al., 2020; Zhang et al., 2022). In the Chinese context, the *Stipa* genus encompasses a diverse array of 23 distinct species, including 6 varieties. Various *Stipa* species, along with their geographic distributions, serve as pivotal criteria in the categorization of grasslands and play a pivotal role in the systematic classification of grassland ecosystems. For instance, within the Mongolian Plateau (MP), the presence of species such as *Stipa gobica*, *S. klemenzii*, or *S. breviflora* distinctly characterizes the desert steppe, while *S. krylovii* and *S. grandis* stand as prominent indicators of the typical steppe. And on the Qinghai-Tibet Plateau (QTP), alpine steppes find their primary constitution in the form of *S. purpurea*, *S. basiplumosa*, and *S. roborowskyi* (Zhou, 1980; Guo et al., 1983; Lu and Wu, 1996; Zhang, 2012; Lu et al., 2018; Qiao, 2019; Li et al., 2020; Mao et al., 2021). Therefore, a comprehensive exploration of the origin and evolutionary trajectory of the *Stipa* genus holds paramount significance in advancing our understanding of Chinese grassland ecology.

The scarcity of fossil evidence initially constrained the investigations on the phylogeny of *Stipa* species (Wang et al., 1975; Thomasson, 1978; Strömberg, 2005). Such investigations primarily relied on morphological traits (Tzvelev, 1976; Tzvelev, 1989; Jacobs and Everett, 1996). Subsequently, researchers obtained additional relevant molecular data, including nuclear ITS intergenic spacers, intergenic spacers (IGS), and chloroplast gene fragments from this genus (Jacobs et al., 2007; Hamasha et al., 2011; Romaschenko et al., 2012; Krawczyk et al., 2017). Previous studies have indicated that *Stipa* species in Central Asia form a monophyletic clade. However, the phylogenetic relationships within the *Stipa* genus remain unresolved (Nobis et al., 2020). Therefore, it is a challenge to comprehend the phylogeny of this genus. Therefore, elucidating the robust phylogenetic relationship within the *Stipa* genus is of great ecological significance for understanding its origin and evolutionary patterns, and grassland development.

Rapid progress in sequencing technologies has provided significantly increased access to extensive genetic information. In recent years, there has been a notable expansion in research methodologies, including the application of complete chloroplast genomes (Krawczyk et al., 2018; Krawczyk et al., 2022), thousands of Single Nucleotide Polymorphisms (SNPs) genotyping using genome-wide (Baiakhmetov et al., 2021b), and the exploration of nucleolar organizing regions (NORs) in the genomic analysis of *Stipa* (Baiakhmetov et al., 2021a). These phylogenetic studies are providing more comprehensive insights than earlier research, resulting in new progress regarding the phylogenetic relationships of species within the *Stipa* genus (Krawczyk et al., 2018; Baiakhmetov et al., 2021b; Krawczyk et al., 2022).

*Stipa*, as the core taxon within the Stipeae, represents a rigorously monophyletic assemblage, divided into two distinct clades. One clade is exclusive to species inhabiting the Himalayan region, while the other encompasses species distributed across various Eurasian territories (Hamasha et al., 2011). A congruent pattern was identified by Peng (2015) in his research (Peng, 2015); however, it’s worth noting that this pattern doesn’t appear to universally hold across all investigations (Krawczyk et al., 2017; Krawczyk et al., 2018; Baiakhmetov et al., 2021b; Krawczyk et al., 2022). While existing methodologies applied to *Stipa* phylogeny have proven effective in elucidating taxonomic relationships at the genus level and beyond, their performance diminishes when exploring relationships below the genus level. Notably, in these studies, the support for internal branches within the *Stipa* phylogenetic tree exhibited certain limitations, with instances of notably weak support. Therefore, there is an imperative need to explore more precise and efficient methods for reconstructing the phylogenetic relationships within the *Stipa* genus, particularly within its relatively underexplored Chinese distribution range.

Compared with genomic sequencing, transcriptome sequencing efficiently provides significant-high-quality data regarding protein-coding genes, making it particularly suitable for studying nonmodel organisms (Wickett et al., 2014; Zimmer and Wen, 2015; Zeng et al., 2017; Baiakhmetov et al., 2021b). Comparative transcriptomics has emerged as an effective method for evolutionary analyses in plants (Cheng et al., 2018; One Thousand Plant Transcriptomes Initiative, 2019; Kapli et al., 2020) and has significantly promoted the identification of phylogenetic relationships among closely related species, as demonstrated in *Cyathophora* (Wang et al., 2017), *Trigonopedia* (Guo et al., 2018), and *Daghestanica* (Xie et al., 2020). Zhang et al. conducted transcriptome analyses of 157 Pooideae species, identified 1234 orthologous genes, and reconstructed the highly supported maximum likelihood (ML) phylogenetic tree using the concatenation method (Zhang et al., 2022). The phylogenetic tree included three species of *Stipa* in China, with branches that received high support. This provides valuable insights into reconstructing the phylogenetic relationships within the *Stipa* genus.

In this study, we have gathered 12 *Stipa* species, representing five of the seven recognized sections within the *Stipa* genus found in China. Notably, these encompass the principal and prevailing species inhabiting diverse *Stipa* grassland ecosystems on the QTP and the MP within Chinese territory. Building upon prior research, which has underscored the relationship between the phylogenetic structure of *Stipa* and the geographical distribution of its constituent species, the primary objective of this paper is to scrutinize the phylogenetic aspects of these 12 *Stipa* species in China. Our approach involves the generation of novel transcriptome data, an invaluable resource enabling an in-depth exploration of the evolutionary history underpinning the *Stipa* in China.

## 2 Materials and methods

### 2.1 Sample collection

**Figure 1:**
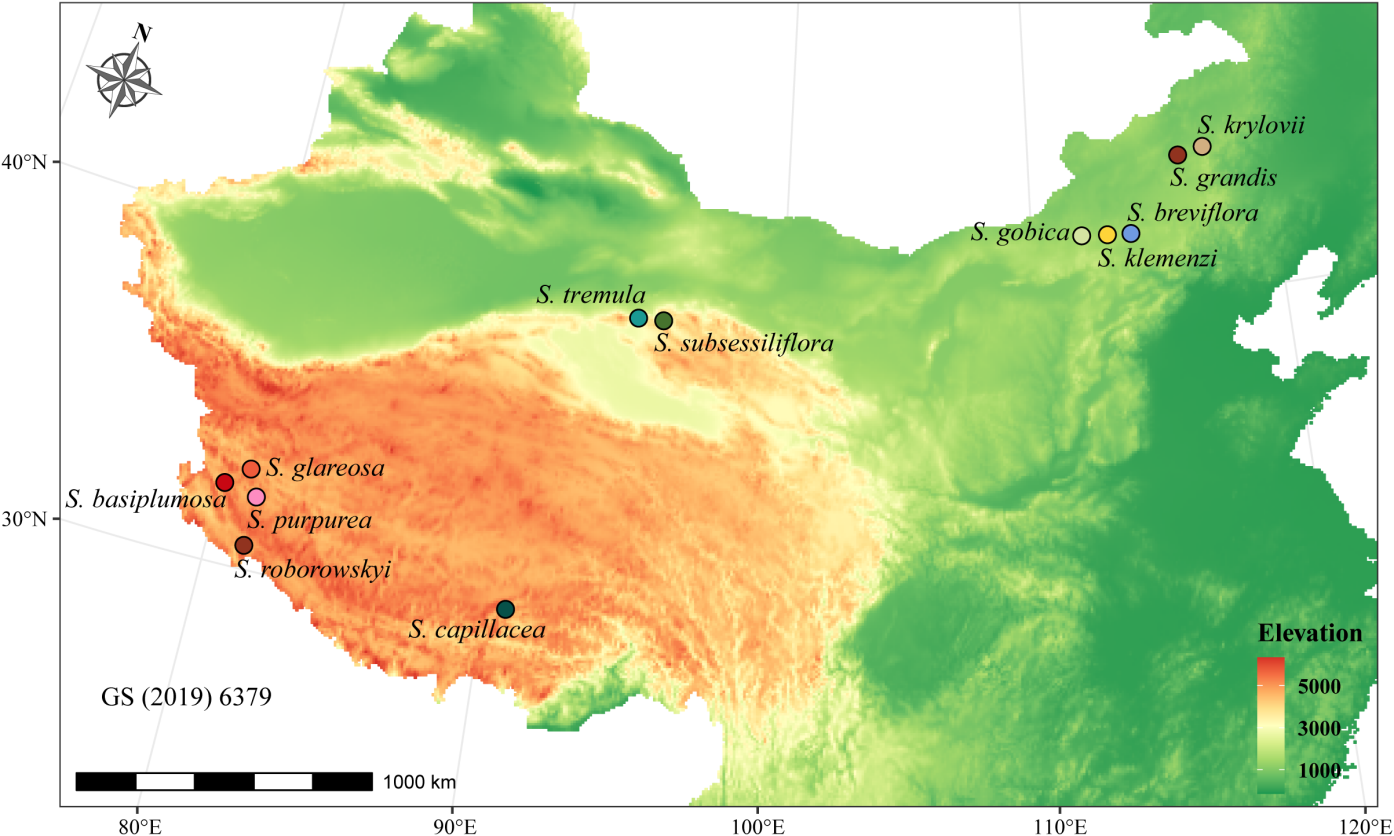
Geographical distribution of sampled *Stipa* species. Each color-matched circle on the map corresponds to a different *Stipa* species, with the names of the species listed nearby.

In total, 12 *Stipa* species were collected from their respective distribution regions from June to August in 2018 and 2019 (**Figure 1** and **Table 1**). At least one specimen of each species was stored in the herbarium of the College of Ecology and Environment, Inner Mongolia University, and the specimens were identified by Prof. Liang, and voucher specimen numbers were in the **Supplementary Data Table S3**. Among them, seven species were high-altitude species (3200–4800 m above sea level) native to the QTP, whereas the other five species were distributed in the relatively low-altitude (1100–1500 m) MP (**Figure 1** and **Table 1**). To ensure the RNA integrity of the samples, we specifically selected the spikelets during the heading period of *Stipa* that were tightly wrapped by bracts and not exposed to air. Five individuals of each species were collected. Their spikelets were immediately placed in liquid nitrogen and stored at −80°C for subsequent RNA extraction.

**Table 1:** Sampling sites of *Stipa* species.

Species	Collection site	Longitude	Latitude	Elevation (m)
*S. basiplumosa* Munro et Hook.f.	QTP_alpine desert steppe	80.63308333	32.37541667	4703
*S. purpurea* Griseb.	QTP_alpine desert steppe	81.36088889	32.18227778	4664
*S. roborowskyi* Roshev.	QTP_alpine steppe	81.33377778	30.70369444	4632
*S. glareosa* P. Smirn.	QTP_alpine desert steppe	80.93722222	32.45052778	4502
*S. capillacea* Keng	QTP_alpine steppe	90.80077778	30.30913889	4318
*S. tremula* (Rupr.) M.Nobis	QTP_alpine steppe	94.34863889	39.34158333	3148
*S. subsessiliflora* (Rupr.) Roshev.	QTP_alpine steppe	94.34231389	39.33923889	2846
*S. breviflora* Griseb.	MP_desert steppe	114.0684306	41.97161944	1502
*S. gobica* Roshev.	MP_desert steppe	112.0586722	42.02148333	1492
*S. klemenzii* Roshev.	MP_desert steppe	113.1091	41.99536389	1449
*S. grandis* P. Smirn.	MP_typical steppe	116.3265111	44.14711667	1118
*S. krylovii* Roshev.	MP_typical steppe	116.3931806	44.31378611	1118

The “Collection Site” column provides insights into the ecological realm of each *Stipa* species, denoted by “QTP” for the Qinghai-Tibet Plateau and “MP” for the Mongolian Plateau, representing their respective steppe habitats.

### 2.2 RNA extraction and transcriptome sequencing

Total RNA was extracted from each sample using ethanol precipitation and CTAB-PBIOZOL reagent as per the manufacturer’s instructions (Hangzhou Bori Technology, Hangzhou, China). The quality and quantity of the extracted RNA were assessed using a NanoDrop spectrophotometer and an Agilent 2100 Bioanalyzer (Thermo Fisher Scientific, MA, USA), respectively. Three samples with an RNA integrity number ≥ 6.5 from each *Stipa* species were selected for constructing the cDNA libraries. mRNA was isolated from total RNA using Oligo (dT) beads and fragmented into short fragments using a fragment buffer at an appropriate temperature. The fragmented RNA was used for the synthesis of the first strand of cDNA using random hexamer primers and reverse transcriptase, followed by the synthesis of the second strand using DNA Polymerase I and RNase H. The double-stranded cDNA was purified using QIAquick PCR Extraction Kit (Qiagen) and subjected to end-repair using an A-Tailing Mix and RNA Index Adapters. The final cDNA library was prepared via PCR amplification with AMPure XP beads. The library was sequenced using the BGISEQ-500 platform (BGI, Shenzhen, China) with paired-end reads of 100 bp.

### 2.3 RNA-seq output quality control and transcriptome *de novo* assembly

The raw reads of each sample were processed to obtain clean reads by removing reads containing potential adaptors, unknown nucleotides (>5%), and low-quality reads (reads with >20% nucleotides with base quality < 15) using SOAPnuke (version 1.4.0) (Chen et al., 2017) and trichromatic (version 0.36) (Bolger et al., 2014). The Quality 20 (Q20), Quality 30 (Q30), and Guanine-Cytosine (GC) content of the clean reads were recorded. The clean reads from the three samples of each *Stipa* species were combined and *de novo* assembled using Trinity (version 2.0.6) (Grabherr et al., 2011) with default parameters. CD-HIT (Fu et al., 2012) was used with a cutoff of 95% similarity for clustering and removing redundant contigs in the assembled transcripts, after which nonredundant transcripts were obtained. The results of the assembly of nonredundant transcripts were evaluated based on the shortest sequence length at 50% of the total sequence length (N50) criteria. Furthermore, TransDecoder (version 5.5.0) was used with default settings to predict coding sequences (CDSs) and protein-coding sequences of the nonredundant transcripts (Kim et al., 2015). Benchmarking Universal Single-Copy Orthologs (BUSCO) (Waterhouse et al., 2017) based on the 4896 poles universal single-copy orthologs (https://busco.ezlab.org/frames/plants.htm), the completeness of the assembly results was assessed. In this analysis, we also wished to detect the number of single-copy orthologs among the 12 *Stipa* species.

### 2.4 Orthologous gene identification and gene annotation

The orthologous gene dataset was constructed among the 12 *Stipa* species by comparing the predicted protein sequences from the nonredundant transcripts with the protein data of *Brachypodium distachyon* downloaded from the National Center for Biotechnology Information (NCBI) (ftp://ftp.ncbi.nlm.nih.gov/genomes/all/GCF/000/005/505/GCF_000005505.3_Brachypodium_distachyon_v3.0). This was performed using bidirectional best BLAST (Version 2.2.25) with an e-value cutoff of 1e^−10^, identity > 20%, and bit score > 40 (Altschul et al., 1997). Only one-to-one orthologous gene pairs between one *Stipa* species and *B. distachyon* were retained. The intersection of these 12 sets of one-to-one orthologous gene pairs was taken to obtain the final one-to-one orthologous gene set (**Supplementary Data Figure S2**). To assess the completeness and quality of the assembly, the protein-coding sequences of *B. distachyon* were used as proxies. BLASTp (with a threshold of 1e^−5^) was used for gene function annotation against the nonredundant protein database (NR) of NCBI and the Swiss-Prot protein database.

### 2.5 Phylogenetic analysis and molecular dating

Multiple sequence alignments of the protein sequences were performed using MAFFT (Katoh et al., 2002) and converted to nucleotide alignments using PAL2NAL (Suyama et al., 2006). Poorly aligned positions and divergent regions were eliminated using Gblocks (http://molevol.cmima.csic.es/castresana/Gblocks_server.html) (Talavera and Castresana, 2007). The filtered nucleotide sequences were translated back to protein sequences. The phylogenetic relationships of the *Stipa* were reconstructed using the concatenation method and several different tree-building strategies as given in the flowchart in **Figure S2**. *B. distachyon* as an outgroup of the *Stipa*. In the RaxML software, the ML tree was constructed after searching for the best model using the built-in command, with the bootstrap set to 1000 replicates (Stamatakis, 2014). In IQtree, the ML tree was built after searching for the evolutionary models for sequence partitions using ModelFinder, with bootstrap set to 1000 replicates (Nguyen et al., 2015; Kalyaanamoorthy et al., 2017). The Bayesian inference (BI) tree was constructed using the Bayesian method, with the best models for sequence partitions found after 200,000 Markov Chain Monte Carlo (MCMC) iterations until significant convergence was achieved (Ronquist et al., 2012).

To estimate the divergence times of the *Stipa* genus, MCMCtree from PAML was used. The mean and 95% highest posterior density (HPD) intervals of divergence times for each node were obtained (Yang, 1997). Considering the lack of fossil records of *Stipa*, we calibrated the divergence times using the divergence time of *B. distachyon* and *Stipa* [27–58 million years ago (Mya)] obtained from the TimeTree project (www.timetree.org/) (Kumar et al., 2017). The tree topology was visualized using the ggtree package in R software (Yu et al., 2017).

## 3 Results

### 3.1 Transcriptome sequencing and basic bioinformatic analysis

A total of 3807.24 million paired-end clean reads were generated from the sequencing data of 380.71 gigabases (Gb) obtained from the 36 *Stipa* samples. The average size of clean reads per sample was 10.58 gigabytes (Gb), with average Q20 and Q30 values of 97.75% and 92.00%, respectively (**Supplementary Data Table S1**).

The assembly process provided nonredundant transcripts for the 12 *Stipa* species, with an average of 227,312 unigenes (≥200 bp) and an average N50 value of 1622 bp (**Table 2** and **Supplementary Data Table 1**). Furthermore, predicted CDSs were obtained, with an average of 87,406 CDSs and N50 values ranging from 1032 to 1152 bp (**Table 1**).

**Table 2:** *De novo* assembly of the sequenced transcriptomes.

Species	Raw reads (Gb)	Clean reads (Gb)	No. of transcripts (≥ 200 bp)	N50	N90	GC	Number of CDSs
*S. basiplumosa*	12.28	10.74	275,666	1522	346	0.47	96,975
*S. purpurea*	12.29	10.67	285,797	1505	350	0.47	97,215
*S. roborowskyi*	12.53	10.80	292,136	1635	355	0.47	104,593
*S. glareosa*	12.43	10.68	223,180	1808	403	0.47	89,111
*S. capillacea*	12.45	10.70	222,139	1739	396	0.47	88,217
*S. tremula*	11.24	10.49	233,783	1605	366	0.48	91,796
*S. subsessiliflora*	12.36	10.94	229,897	1541	346	0.46	86,337
*S. breviflora*	11.24	10.49	177,458	1858	430	0.47	80,034
*S. gobica*	10.93	10.28	214,119	1603	342	0.47	84,886
*S. klemenzi*	11.18	10.51	225,556	1477	330	0.47	80,357
*S. grandis*	11.14	10.34	199,731	1796	401	0.48	84,491
*S. krylovii*	11.06	10.27	148,279	1374	342	0.48	64,864

The values presented under “Raw reads” and “Clean reads” are the averaged values from three biological replicates of each species. The abbreviation “Gb” stands for giga base, and “bp” represents base pair.

The BUSCO analysis indicated the completeness of the transcriptome assembly, which was approximately 84% on average across the 12 *Stipa* species (**Supplementary Data Figure S1**).

Annotation results using *B. distachyon* as a reference revealed that 99% of the orthologous genes had hits in the NR database and 75% in the SwissProt database. In total, 73% of the orthologous genes were assigned to Gene Ontology categories, and 48% had matches in the KEGG pathway database (data not presented). These results indicated that the transcriptomes of the *Stipa* species were well assembled and relatively complete, providing high-quality data for further comparative transcriptome analyses.

### 3.2 Identification of orthologous genes

Using the bidirectional best BLAST analysis, we identified one-to-one orthologous genes in the protein-coding sequences of *B. distachyon* and *Stipa* species, obtaining 10,717 shared genes among the 12 *Stipa* species. By increasing the BLAST parameter identity to 30%, 9418 genes were retained. Subsequently, these genes were further filtered based on sequence saturation and multiple alignment analysis, to obtain 9397 genes forming the “one2_orthologous” gene set. Further, 2300 orthologous genes with high homology (90%) were extracted. Additionally, from the set of 4896 single-copy orthologous genes retrieved from the BUSCO database, 62 single-copy orthologous genes shared among the 12 species were identified, and this set was designated as the “SCG_orthologous” gene set. Finally, the three gene sets were combined to obtain three supergenes, with lengths 7,637,640, 2,314,182, and 118,167 bp, respectively, for the phylogenetic analysis of the *Stipa* genus (**Supplementary Data Figure S2**).

### 3.3 Phylogenetic analysis

**Figure 2:**
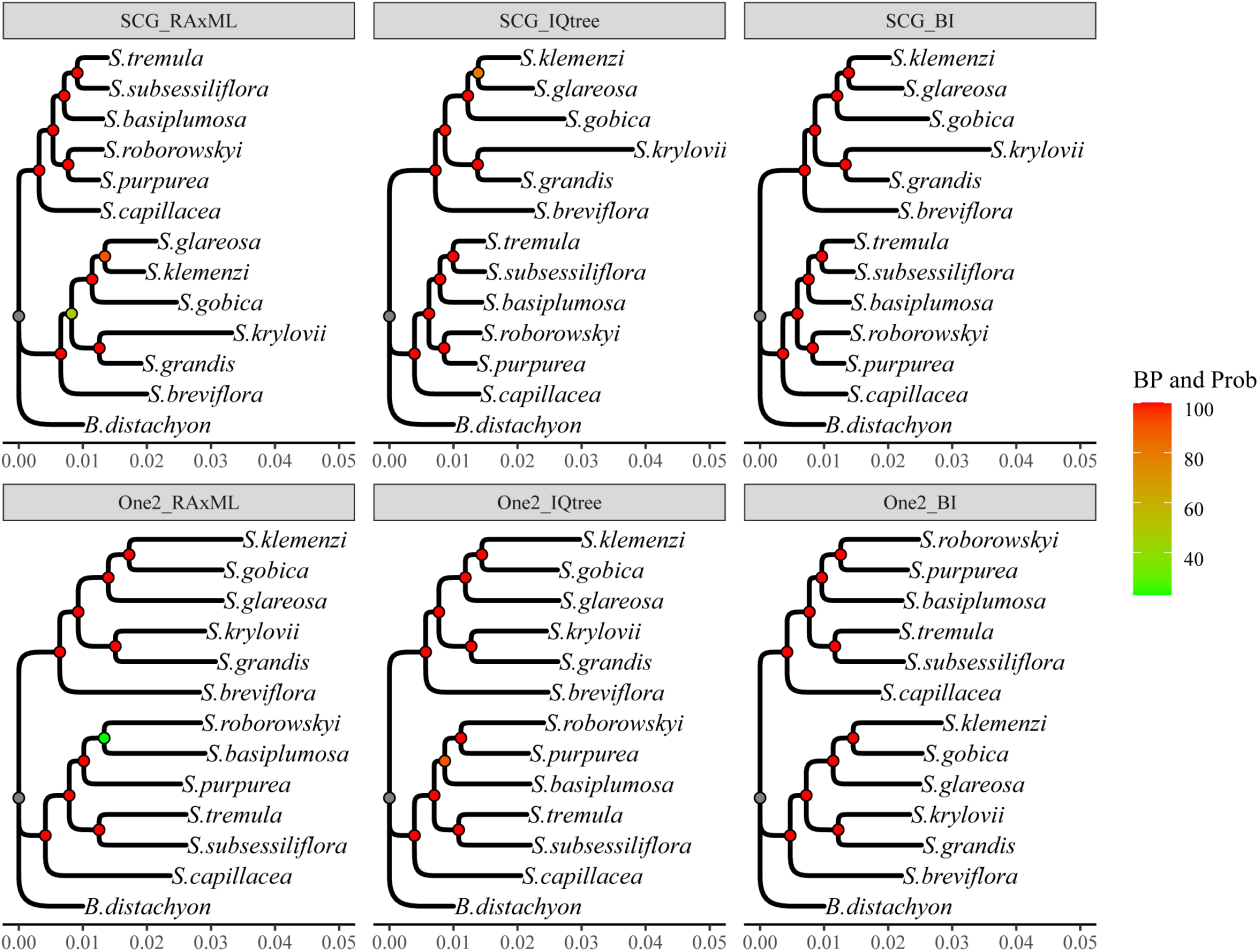
Phylogenetic trees constructed via varied strategies. The tree’s top name corresponds to the tree in the flowchart in **Supplementary Data Figure S2**. The Bayesian Inference (BI) tree carries a node support probability (Prob), equivalent to 100% bootstrap values (BP) in the Maximum Likelihood (ML) tree. Species names appear on the right side of the branch tips. The values below the tree represent branch lengths, which have been shortened for the outgroup *Brachypodium distachyon* for readability.

Considering the computational time and resource constraints, we initially adopted various tree-building strategies for the analysis of the SCG_orthologous gene set. First, using the RaxML software with default parameters, the best model was searched. Subsequently, a maximum likelihood (ML) tree was constructed (**Supplementary Data Figure S2**). In the resulting tree, only one branch had a low bootstrap value (BP = 47%), and others had BP > 90% (SCG_RaxML tree) (**Figure 2**). Next, we identified the optimal evolutionary model using ModelFinder and constructed an ML tree with IQtree (Edge-linked partition model) (**Supplementary Data Figure S2**). The results revealed that the BP for the tree topology was all above 80%, with the majority being 100% (SCG_IQtree) (**Figure 2**). Finally, using the MrBayes Bayesian method, a BI tree was obtained that exhibited high stability, with each branch having a support value of 100% (SCG_BI tree) (**Figures S2** and **2**). Therefore, the SCG_orthologous supergenes provided consistent topological structures in the evolutionary trees generated by different software and methods, exhibiting high stability (**Figure 2**).

**Figure 3:**
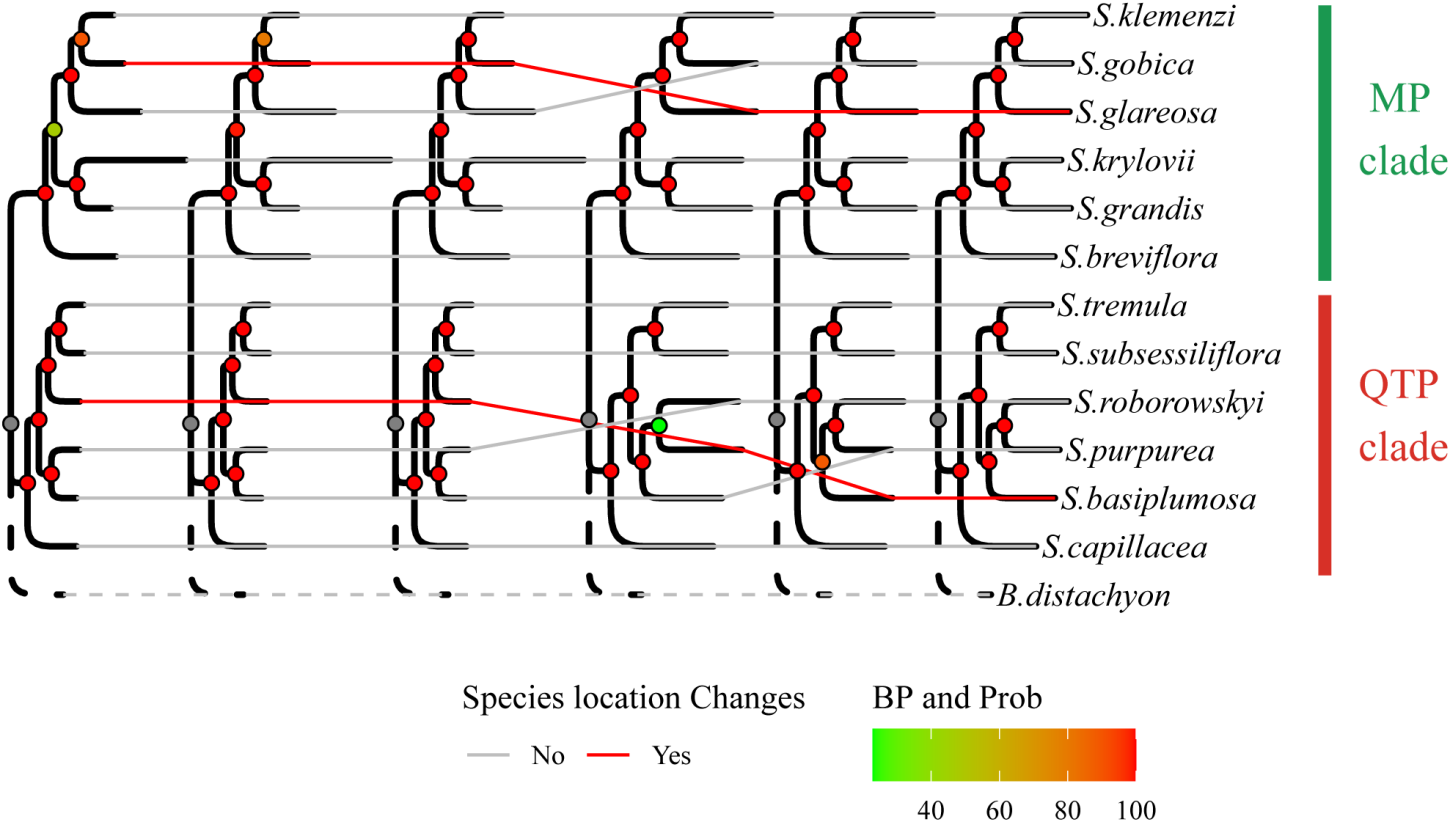
Evolutionary shifts in species placement within the phylogenetic tree. The arrangement of species positions in the depicted phylogenetic tree described illustrates intriguing evolutionary changes. The sequence of the six phylogenetic trees from left to right reflects the arrangement of the six trees displayed in **Supplementary Data Figure S2**.

The phylogenetic tree was constructed using two distinct datasets of one2_orthologous genes, comprising 9397 and 2300 sequences, respectively. Notably, the one2_RaxML tree exclusively consisted of 9397 immediate homologous genes, whereas the one2_IQ tree and one2_BI tree were formed using a partitioning model and Bayesian approach, respectively, with the 2,300 selected genes (**Supplementary Data Figure S2**). The results revealed remarkable stability and similarity in the topologies of these three trees, exhibiting only minor discrepancies, particularly regarding the relative position of *S. basiplumosa*. Within both one2_IQtree and one2_BI trees, *S. basiplumosa* demonstrated a consistent and robust relationship with *S. purpurea* and *S. roborowskyi*, supported by a high BP of 89% and a probability of 100%. However, in the one2_RaxML tree, the branch containing *S. basiplumosa* exhibited a significantly lower self-expansion value and merely 23% BP (**Figure 2**). Interestingly, the relative position of *S. basiplumosa* with neighboring species differed between the topology of one2_orthologous and SCG_orthologous (**Figure 3**).

Specifically, in SCG_orthologous, *S. basiplumosa* displayed a closer association with *S. tremula* and *S. subsessiliflora* (BP = 100%), whereas in one2_orthologous, it appeared more closely related to *S. purpurea* and *S. roborowskyi* (BP = 89%). However, the exclusion of *S. basiplumosa* did not alter the positioning of these species within the one2_orthologous tree. Notably, the position of *S. glareosa* remained consistent within the one2_orthologous topology [(*S. glareosa*, (*S. gobica*, *S. klemenzi*)), BP = 100%], although it slightly deviated from the SCG_orthologous tree [(*S. gobica*, (*S. glareosa*, *S. klemenzi*)), BP = 80% or 90%, prob = 100%] (**Figures 2** and **3**).

In summary, the phylogenetic relationships constructed from the two gene sets were generally consistent and reflected a common feature, that is, the internal divergence of the *Stipa* genus into two distinct branches (BP = 100%). These two branches were respectively represented by the QTP species, namely, *S. capillacea*, *S. purpurea*, *S. roborowskyi*, *S. tremula*, and *S. subsessiliflora* forming the QTP clade (QTP_clade) and by the MP species, namely, *S. breviflora*, *S. gobica*, *S. klemenzi*, *S. krylovii*, and *S. grandis*, along with a QTP species *S. glareosa*, forming the MP clade (MP_clade) (**Figure 3**).

### 3.4 Estimation of divergence time

**Figure 4:**
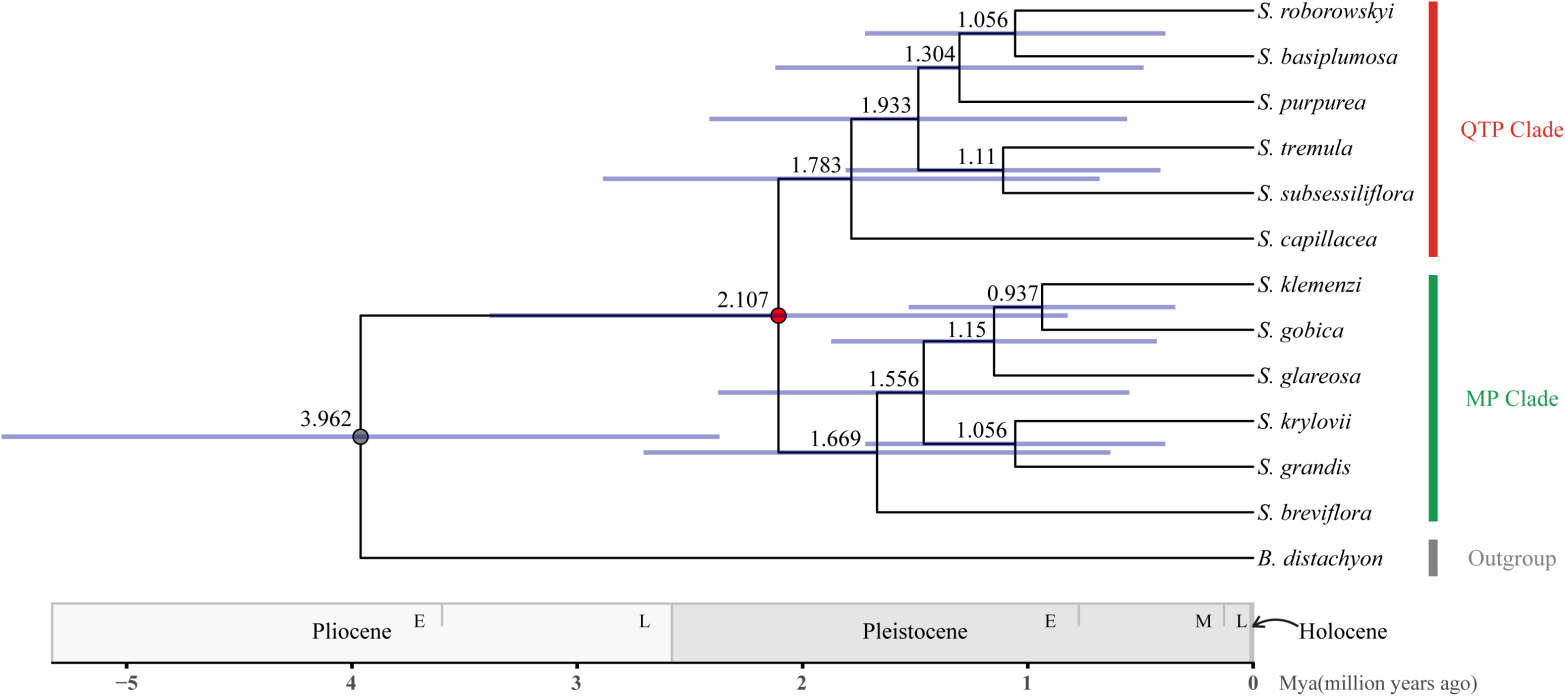
The chronogram of divergence times in the *Stipa* genus. The average age (above branches) and their 95% confidence intervals (expressed in blue bars) were depicted near various nodes in the *Stipa* genus phylogeny. Geological timescales are provided below the tree diagram.

Molecular clock analysis revealed that the divergence between the *Stipa* genus and *B. distachyon* occurred approximately 3.962 Mya (95% CI: 2.486–5.672 Mya). Within the *Stipa* genus, the internal diversification began approximately 2.107 Mya (95% CI: 1.101–3.668 Mya), and the QTP_clade and MP_clade diverged sequentially during 0.45 Mya. The QTP_clade exhibited continuous diversification within the time range of 1.783–1.056 Mya, whereas the MP_clade initiated diversification during the overlapping period of 1.669–0.937 Mya. Eventually, the two clades completed species diversification synchronously within 0.846 Mya (**Figure 4**).

In the MP_clade, the desert grassland species *S. breviflora* diverged first, followed by *S. glareosa*, *S. gobica*, and *S. klemenzi*. Finally, the typical steppe species *S. krylovii* and *S. grandis* diverged. In the QTP_clade, the alpine meadow species *S. capillacea* diverged first, followed by the western QTP desert grassland species *S. basiplumosa*, *S. purpurea*, and *S. roborowskyi*. Lastly, the species located in the northern Qilian Mountains, *S. tremula* and *S. subsessiliflora*, diverged.

## 4 Discussion

### 4.1 The application of transcriptome in constructing phylogenetic relationships of *Stipa*

Plastid genes, particularly chloroplast genes, serve as a stable marker system in phylogenetic analysis because of their uniparental inheritance and high conservation (Orton et al., 2021). However, a study on 19 *Stipa* species revealed that the highly similar chloroplast genome sequences of this genus posed a certain interference in phylogenetic analysis (Krawczyk et al., 2018). On the other hand, small variation information carried by a few nuclear genes limited the extraction of sufficient genetic variations and the obtaining of reliable analytical results (Zimmer and Wen, 2015). A few gene fragments may lead to suboptimal saturation of the constructed phylogenetic trees (Cornwell and Nakagawa, 2017; Zeng et al., 2017). Therefore, in the research of *Stipa* species, these two are the primary limiting factors for obtaining high-confidence phylogenetic relationships (Romaschenko et al., 2010; Hamasha et al., 2011; Romaschenko et al., 2012; Peng, 2015; Krawczyk et al., 2017; Krawczyk et al., 2018; Krawczyk et al., 2022). Krawczyk’s research identified a region proximal to the 26S nrDNA, specifically within the nuclear rRNA intergenic spacer region (IGS), as one of the most phylogenetically informative areas among *Stipa* species. Their phylogenetic tree, which encompassed 35 *Stipa* species, marked the first systematic tree for the *Stipa* genus that exhibited well-resolved evolutionary branches. However, it’s noteworthy that only 19 branches garnered support levels exceeding 50% (Krawczyk et al., 2017). Similarly, Baiakhmetov constructed phylogenetic relationships for 14 *Stipa* species using nucleolar organizing regions (NORs), and their findings also indicated relatively low support in the resulting tree (Baiakhmetov et al., 2021b). It appears that there is a paradox in *Stipa* genus phylogenetic research. When researchers aim to enhance the clarity of branches in the phylogenetic tree, as discussed above, they often encounter challenges in achieving higher support levels.

Consequently, Krawczyk et al. suggested the incorporation of more nuclear genes in the phylogenetic analysis of the *Stipa* genus (Krawczyk et al., 2018). Later, Baiakhmetov employed a genome-wide approach to obtain thousands of SNP genotypes for a phylogenetic analysis of a population comprising five *Stipa* species. The results indicated that fewer than half of the branches could be supported with a value of ≥0.8 (Baiakhmetov et al., 2021b). In our study, the high-confidence phylogenetic trees of *Stipa* were obtained from dozens and thousands of protein-coding genes, with almost 100% support for all branches (**Figure 2**). This complements the low resolution of the previous phylogenetic tree of *Stipa*.

This was possible because of the rapid development of sequencing technology. Rapid analysis of numerous nuclear genes through transcriptome sequencing has been widely used in reconstructing phylogenetic relationships in plants (One Thousand Plant Transcriptomes Initiative, 2019), providing good results across green plant groups and higher taxonomic units (Zeng et al., 2014; Guo et al., 2018; Zhang et al., 2022). Nevertheless, when using a large amount of gene data for phylogenetic analysis, the isolation of gene sets and tree-building strategies can influence the final results (Blair and Murphy, 2011; Zeng et al., 2014; Cornwell and Nakagawa, 2017; Zeng et al., 2017). In this study, transcriptome sequencing, assembly, and screening were conducted on 12 *Stipa* species, the majority of which are dominant or key species of *Stipa* on the grasslands found in the QTP and MP. Through meticulous analysis of orthologous genes within the transcriptome data, two sets containing high-quality orthologous genes (2300 and 9397, respectively) were obtained. These genes were subjected to phylogenetic analysis using various tree-building strategies (**Supplementary Data Figure S2**). The results indicated that the orthologous genes of *Stipa* species exhibited low sensitivity to different tree-building methods (**Figure 2**), thus potentially providing stable phylogenetic relationships.

### 4.2 Identification of two geographically related clades within the *Stipa* genus

Although the lack of data is limiting the phylogenetic studies on *Stipa*, we can still obtain some insights based on available studies. Hamasha et al. (2011) conducted a phylogenetic analysis of the Stipeae tribe using ITS and ribosomal genes and reported that several *Stipa* species from the QTP, including *S. basiplumosa*, *S. rohmooiana*, *S. subsessiliflora*, and *S. purpurea*, formed a distinct branch within the *Stipa* genus. Another larger branch of the *Stipa* genus consisted of *S. breviflora*, *S. baicalensis*, *S. gobica*, and other species from the Tianshan Mountains, MP, and other regions (Hamasha et al., 2011). Peng (2015) constructed a phylogenetic tree of Chinese *Stipa* species using ITS and chloroplast genes and reported that the *Stipa* genus primarily differentiated into two clades. One clade consisted of *Stipa* species from the Sect. *Pseudoptilagrostis* Tzvel. and some species of the Sect. *Barbatae* Junge was primarily distributed in the QTP, whereas the other clades included *Stipa* species from the Sect. *Leiostipa* Dum, Sect. *Stipa*, Sect. *Smirnovia* Tzvel, and the remaining species of the Sect. *Barbatae* Junge (Peng, 2015). To maintain consistency with the previous discussions, we referred to these two clades as the “QTP_clade” and “MP_clade”, representing the major distribution areas of *Stipa* species in the QTP and MP, respectively. In the aforementioned studies, the nodes supporting the separation of the two clades had relatively high BP, which was consistent with our finding that the BP for the QTP and MP clades was 100% (**Figure 2**). However, some differences in the internal relationships within each clade were observed in our previous studies. In a study by Hamasha, *S. capillacea* from the QTP were clustered within the non-QTP branch. In a study by Peng, *S. capillacea* was assigned to the QTP clade, whereas *S. purpurea* was classified within the MP clades. In our study, these species were clustered within the QTP clades (**Figures S2** and **2**).

Furthermore, within the taxonomic realm of the Sect. *Smirnovia* Tzvel. [comprising species such as *S. glareosa*, *S. gobica*, and *S. klemenzii*, primarily inhabiting the desert steppe of the MP (BP > 89%)] and the Sect. *Leiostipa* Dum. [comprising *S. krylovii* and *S. grandis* (BP = 100%), dominantly found in the typical steppe of Mongolia], we observed stable clustering that adheres to traditional taxonomic classifications based on distinctive traits (Nobis et al., 2020). However, *S. breviflora*, a member of Sect. *Barbatae* Junge, typically does not cluster within this section but is placed in non-QTP branches (Peng, 2015; Nobis et al., 2020; Baiakhmetov et al., 2021b; Krawczyk et al., 2022). Similarly, other members of Sect. *Barbatae* Junge, including *S. tremula*, *S. roborowskyi*, and *S. purpurea*, were grouped based on morphological characteristics (Hamasha et al., 2011; Peng, 2015), then clustered with Sect. *Pseudoptilagrostis* Tzvel., containing *S. basiplumosa* and *S. subsessiliflora*, forming a coherent clade on the QTP (**Figures 2** and **3**). These findings are consistent with a previous study (Hamasha et al., 2011; Peng, 2015), highlighting the importance of our results in providing highly supported and reliable data for resolving the developmental relationships among *Stipa* species.

In the context of phylogenetic analysis based on morphological traits, members of the *Barbatae* section typically manifest as small branches comprising multiple taxa. These branches subsequently intertwine within the *Pseudoptilagrostis*, *Smirnovia*, and *Leiostipa* sections (Nobis et al., 2020). In contrast, the members of *Smirnovia* and *Leiostipa* sections often form distinct and stable clusters (Romaschenko et al., 2012; Baiakhmetov et al., 2021b). This observation implies that taxonomic traits from different sections contribute variably to the phylogenetic signal. It also corroborates earlier findings, suggesting that many crucial classification traits within Stipa lack robust phylogenetic signals (Romaschenko et al., 2012).

### 4.3 Geological and climate changes may drive species diversification in the *Stipa* genus

The phytolith data from the Eocene-Miocene period in the North American interior provides evidence that the Stipeae appeared in the early Oligocene (34 Mya) and transitioned from closed habitats to open environments. However, it was not until the Late Oligocene or Early Miocene (7-11 million years ago) that the Stipeae became ecologically dominant in North America alongside other grasses (Strömberg, 2005). Molecular evidence suggests that approximately 30 Mya, the Stipeae underwent diversification into two major clades (New World and Old World) and experienced a period of stable temperatures (Romaschenko et al., 2010; Romaschenko et al., 2012; Zhang et al., 2022). During the middle Miocene (approximately 14 Mya), a rapid cooling occurred, leading to the differentiation of several genera including *Stipa* (Zhang et al., 2022). Nevertheless, our findings indicate that the estimated time of differentiation between *Brachypodium distachyon* and *Stipa* was approximately 3.962 Mya (95% CI: 2.486-5.672 Mya) (**Figure 4**). This value notably deviates from this fossil-based estimation.

We hypothesize that the considerable disparity in our results could be attributed to the relatively broad calibration time frame we employed. Specifically, we utilized a calibration range of 27 to 58 Mya for the separation of the Stipeae tribe and the *Brachypodium distachyon*, aligning it with the divergence point between the outgroup and *Stipa*. In comparison to the methodologies of Baiakhmetov et al. (2021b) and Zhang et al. (2022), which encompassed a more diverse array of calibration points and strategies, these studies achieved a higher degree of accuracy. In the investigation conducted by Baiakhmetov, a dual dataset approach was utilized for calibration (Baiakhmetov et al., 2021b). One dataset indicated a divergence between Brachypodium and Oryza approximately 38 to 48 Mya, while the other dataset estimated the emergence of *Stipa* at 33 to 39 Mya. Their results suggest that *B. distachyon* and *Stipa* diverged around 32.77 Mya. In another comprehensive study utilized a multifaceted approach involving three calibration strategies and a multitude of calibration points, encompassing various species within the Pooideae (Zhang et al., 2022). Their results suggested the appearance of Stipeae around 30 Mya, with *Stipa* emerging approximately 12.7 Mya. These studies, which utilized a more diverse set of calibration points and strategies, achieved higher precision and demonstrated closer alignment with empirical data. Hence, it becomes clear that the inclusion of multiple, highly accurate calibration points and strategies is imperative for attaining precise estimates of divergence times in our research endeavors.

Currently, molecular clock analyses estimate the origin of the *Stipa* genus as the middle Miocene to early Pliocene (Baiakhmetov et al., 2021a; Baiakhmetov et al., 2021b; Zhang et al., 2022). Our results show that *Stipa* emerged and began to differentiate at 2.107 Mya (95% CI: 1.101-3.668; **Figure 4**), which is slightly later than the current results. The discrepancy between the estimated molecular clock origin time and the earliest fossil record of *Stipa* from the North American interior (23 Mya, Elias, 1942; Thomasson, 1978; Thomasson, 1985; Strömberg, 2005) may be due to several factors. One possibility is the limited number of *Stipa* species used in both previous studies and our own. Another factor could be the extinction of ancient *Stipa* species, leading to a delay in the molecular clock estimation of the origin compared to the fossil record. It’s important to note that the North American concept of *Stipa* encompasses a broader spectrum compared to the Eurasian concept (Hamasha et al., 2011; Romaschenko et al., 2012; Nobis et al., 2020), including numerous species with substantial morphological differences from their Eurasian counterparts (Romaschenko et al., 2012). Therefore, the fossil records from North America may not provide a comprehensive representation of Eurasian *Stipa*.

Currently, the fossil records in Eurasian geological strata still lack substantial evidence regarding the presence of *Stipa*. However, according to pollen records of herbaceous plants in Chinese geological strata, there appears to be a gradual increase in the abundance of herbaceous plants, possibly including *Stipa*, during the early Pliocene to Pleistocene period (Jiang and Ding, 2009; Deng et al., 2019; Spicer et al., 2020). This observation aligns reasonably well with the patterns we have observed in our research.

Our study suggested that the *Stipa* genus underwent rapid diversification from the early Pliocene to the Pleistocene (3.962–0.937 Mya; **Figure 4**). This finding is consistent with previous studies based on morphological traits (Tzvelev, 1976; Tzvelev, 1989; Linder et al., 2018) and pollen records of herbaceous plants (Jiang and Ding, 2009; Zhang, 2012; Deng et al., 2019). The divergence time of *Stipa* (4.39-0.16 Mya) obtained by Baiakhmetob through nucleolar organizing regions (NORs) was also extremely similar, although it received weak support from the phylogenetic tree (Baiakhmetov et al., 2021b). The notable features of the Pliocene include a rapid global temperature decline, extensive expansion of the Arctic ice cap (Goldner et al., 2014), the final significant uplift of the QTP (Spicer et al., 2020), and glacial–interglacial oscillations in the Pleistocene (Liu et al., 2001). Although the uplift history of the QTP is still debated (Wang et al., 2014; Spicer et al., 2020), its uplift has been confirmed to intensify the Asian monsoon system and inland aridification, promoting biodiversity in the region (Dupont-Nivet et al., 2007; Favre et al., 2015; Sun et al., 2017; Li et al., 2021; Mao et al., 2021; Wu et al., 2022). The Pleistocene climatic oscillations created conducive circumstances for the proliferation of species specialized for cold habitats, resulting in the emergence of numerous endemic vicariants in Central Asia and the western Pamir-Alai Mountains, which serve as significant centers for species diversification. Specifically, *Stipa gracilis* and *S. zeravshanica* exemplify this phenomenon (Vintsek et al., 2022). These species exhibit distinct characteristics that align with the “interglacial refugia model,” wherein populations undergo expansions during glacial periods followed by abrupt demographic contractions during interglacial periods (Bennett and Provan, 2008). The spikelet traits of the *Stipa* genus may be associated with adaptation to arid and monsoonal environments. For instance, the dorsal awn of the spikelet can aid plants in attaching to animals and enhancing seed burial in the soil and can facilitate long-distance dispersal aided by monsoonal systems; moreover, a fur-like hairy ovary indumentum can protect the ovary and increase drought resistance (Tzvelev, 1989; Strömberg, 2011; Linder et al., 2018).

During the early Pleistocene (2.107 Mya), the *Stipa* genus diverged into two clades, consisting of native species from the QTP and MP, respectively, and completed the speciation process during the middle Pleistocene (1.783–0.937 Mya, **Figure 4**). This divergence could be attributed to the geographical isolation caused by the uplift of the Qilian Mountains in northern QTP (Rowley and Currie, 2006; Spicer et al., 2020; Wu et al., 2022). Additionally, the glacial–interglacial cycles in the Pleistocene further intensified aridity in the northwestern inland regions of China (An et al., 2006), range shifts of many taxa including *Stipa* (Vintsek et al., 2022), leading to adaptive differentiation within the two clades and gradual accumulation of genetic distance. During glaciations in the Pleistocene, a unified, extensive ice cap covering all land surfaces did not form in China, and glaciation only occurred on mountain peaks. This made the central QTP and MP a place for the development of numerous Central Asian flora and fauna (Mao et al., 2021). The evolutionary complexity and diverse habitats of the QTP and MP provided multiple ecological niches for the survival and proliferation of *Stipa* plants (Guo et al., 1983; Liu et al., 2014; Qiao, 2019). Many researchers believe that cooling during the Pliocene significantly promoted the origin and expansion of temperate biotic communities (particularly for the subfamily Pooideae), which benefitted from key morphological innovations and genome duplication events in a spikelet (Sandve et al., 2011; Vigeland et al., 2013; McKain et al., 2016; Linder et al., 2018). Whole-genome duplication events have been observed exclusively in the New World clade of Stipeae, specifically in genera such as *Austrostipa* (Tkach et al., 2021), while no such events have been detected in Old World *Stipa* genera (Zhang et al., 2022). *Stipa* may have acquired allopolyploidy through hybridization with distantly related diploid species (Tzvelev, 1989; Krawczyk et al., 2018; Nobis et al., 2019; Baiakhmetov et al., 2020; Baiakhmetov et al., 2021a; Baiakhmetov et al., 2021b; Nobis et al., 2022), thereby obtaining and retaining beneficial genes related to stress response or reproductive development.

### 4.4 Evolution of the *Stipa* genus has always been accompanied by the development of grasslands

Since the Middle Miocene, the evolution of herbaceous plants has outpaced that of woody plants due to the habitat provided by mountainous movements and cooling climate, making the western region of China a center for the development of herbaceous plants. From the Middle Miocene onward, the number and variety of herbaceous plants significantly increased, reaching the peak during the Pliocene. During this period, grasslands or grassy areas expanded southeastward, and a modern grassland distribution pattern emerged in China during the Early Pliocene, extending from northwest to southeast (approximately 14–6 Mya) (Jiang and Ding, 2009; Strömberg, 2011; Lu et al., 2018). *Stipa* began to diversify at a very rapid rate since its emergence in the Pliocene (**Figure 4**). Various plants from grassland, including those from *Stipa*, as well as Asteraceae, Liliaceae, Leguminosae, and Poaceae, began to develop. This led to the formation of *Stipa* steppes dominated by *Stipa* species in the late Pliocene, which were widely distributed in the QTP and MP regions (Wang, 1997; Li, 1999; Strömberg, 2011; Li et al., 2015; Deng et al., 2019).

## 5 Conclusion

Using various transcriptomic datasets, we evaluated and compared various phylogenetic analysis methods to reconstruct the high-resolution phylogenetic relationships within the *Stipa* genus. This study provided important clues to the phylogeny and evolutionary history of *Stipa*. The results revealed that *Stipa*, as an early classification unit within the Stipeae tribe, originated from the Middle Miocene to the Early Pliocene. During the Early Pleistocene, it diversified into two clades in China, namely, the QTP clade and the MP clade, consisting of native *Stipa* species distributed on the QTP and MPs, respectively. It underwent rapid parallel diversification within the Pleistocene. Geological events and climate changes during this period likely acted as external driving factors for the diversification of *Stipa*. Additionally, the characteristic awned inflorescence of *Stipa* and hybridization events may have provided an intrinsic genetic basis for its adaptive evolution. The evolution of *Stipa* was accompanied by the development of grassland landscapes, and *Stipa* species gradually evolved into the dominant and widely distributed species on grasslands.

## Data availability statement

The data presented in the study are deposited in the NCBI repository, with BioProject numbers PRJNA1014579, PRJNA1014757, PRJNA1014596, PRJNA1014658, PRJNA1014760, PRJNA1014801, PRJNA1014811, PRJNA1014756, PRJNA1014755, PRJNA1014613, PRJNA1014758, PRJNA1014800, with specific details provided in **Supplementary Table S2**.

## Author contributions

NS: Data curation, Formal Analysis, Investigation, Validation, Visualization, Writing – original draft, Writing – review & editing. ZL: Formal Analysis, Funding acquisition, Investigation, Project administration, Supervision, Writing – review & editing. QS: Investigation, Writing – review & editing. YH: Investigation, Writing – review & editing. LT: Investigation, Writing – review & editing. YW: Investigation, Writing – review & editing. XL: Investigation, Writing – review & editing. YS: Investigation, Writing – review & editing. JZ: Investigation, Writing – review & editing. JP: Investigation, Writing – review & editing. LW: Investigation, Writing – review & editing. ZD: Conceptualization, Formal Analysis, Funding acquisition, Investigation, Project administration, Supervision, Writing – original draft, Writing – review & editing. CL: Conceptualization, Funding acquisition, Investigation, Project administration, Supervision, Writing – review & editing.
