# Supplementary material for: Elucidation of the evolutionary history of Stipa in China using comparative transcriptomic analysis
Source: Front Plant Sci. 2023 Nov 28;14:1275018. doi: 10.3389/fpls.2023.1275018 (PMC10751131; doi:10.3389/fpls.2023.1275018)
Supplement: Supplementary file 1 [file DataSheet_1.docx]

***Supplementary Material***

**Elucidation of the Evolutionary History of Stipa in China Using Comparative Transcriptomic Analysis**

**Na Sha^†^ , Zhiyong Li^†^ , Qiang Sun, Ying Han, Li Tian, Yantao Wu, Xing Li Yabo Shi, Jinghui Zhang, Jiangtao Peng, Lixin Wang, Zhenhua Dang, Cunzhu Liang**

***Correspondence:** Zhenhua Dang: zhdang@imu.edu.cn

# Supplementary Figures and Tables

**Supplementary Tabel S1** RNA-seq output and quality control.

| Sample | Raw Reads(Gb) | Clean Reads(Gb) | Q20(%) | Q30(%) | Ratio(%) |
| --- | --- | --- | --- | --- | --- |
| *S. basiplumosa*_1 | 12.202 | 10.52 | 96.78 | 89.02 | 86.18 |
| *S. basiplumosa*_2 | 12.202 | 10.68 | 96.89 | 89.36 | 87.55 |
| *S. basiplumosa*_3 | 12.442 | 11.01 | 96.88 | 88.94 | 88.46 |
| *S. purpurea*_1 | 12.202 | 10.6 | 96.74 | 89.01 | 86.83 |
| *S. purpurea*_2 | 12.451 | 10.68 | 96.44 | 88.12 | 85.8 |
| *S. purpurea*_3 | 12.202 | 10.73 | 96.83 | 88.85 | 87.96 |
| *S. roborowskyi*_1 | 12.202 | 10.59 | 97.19 | 89.79 | 86.83 |
| *S. roborowskyi*_2 | 13.198 | 11.04 | 96.76 | 88.86 | 83.61 |
| *S. roborowskyi*_3 | 12.196 | 10.77 | 96.95 | 89.19 | 88.3 |
| *S. glareosa*_1 | 12.451 | 10.71 | 96.75 | 88.99 | 85.98 |
| *S. glareosa*_2 | 12.397 | 10.6 | 96.55 | 88.47 | 85.5 |
| *S. glareosa*_3 | 12.451 | 10.72 | 96.65 | 88.72 | 86.06 |
| *S. capillacea*_1 | 12.451 | 10.61 | 96.75 | 88.59 | 85.17 |
| *S. capillacea*_2 | 12.451 | 10.79 | 97 | 89.34 | 86.65 |
| *S. capillacea*_3 | 12.451 | 10.71 | 96.89 | 88.98 | 86.02 |
| *S.* *tremula* _1 | 11.16 | 10.42 | 98.59 | 94.78 | 93.38 |
| *S.* *tremula* _2 | 11.159 | 10.42 | 98.66 | 95.2 | 93.39 |
| *S.* *tremula* _3 | 11.407 | 10.63 | 98.54 | 94.58 | 93.2 |
| *S. subsessiliflora*_1 | 12.449 | 10.96 | 96.96 | 89.11 | 88.07 |
| *S. subsessiliflora*_2 | 12.442 | 11.02 | 96.84 | 88.75 | 88.6 |
| *S. subsessiliflora*_3 | 12.201 | 10.85 | 96.96 | 89.06 | 88.95 |
| *S. breviflora*_1 | 11.173 | 10.5 | 98.81 | 95.56 | 93.99 |
| *S. breviflora*_2 | 11.174 | 10.49 | 98.73 | 95.27 | 93.9 |
| *S. breviflora*_3 | 11.379 | 10.49 | 98.58 | 94.62 | 92.19 |
| *S. gobica*_1 | 10.924 | 10.27 | 98.76 | 95.25 | 93.98 |
| *S. gobica*_2 | 10.927 | 10.28 | 98.63 | 94.79 | 94.07 |
| *S. gobica*_3 | 10.927 | 10.29 | 98.81 | 95.53 | 94.17 |
| *S. klemenzi*_1 | 11.176 | 10.49 | 98.81 | 95.73 | 93.9 |
| *S. klemenzi*_2 | 11.175 | 10.51 | 98.73 | 95.19 | 94.07 |
| *S. klemenzi*_3 | 11.179 | 10.52 | 98.75 | 95.33 | 94.13 |
| *S. grandis*_1 | 11.147 | 10.35 | 98.53 | 94.47 | 92.88 |
| *S. grandis*_2 | 11.14 | 10.32 | 98.55 | 94.57 | 92.66 |
| *S. grandis*_3 | 11.144 | 10.34 | 98.58 | 94.75 | 92.77 |
| *S. krylovii*_1 | 10.898 | 10.12 | 98.6 | 94.91 | 92.9 |
| *S. krylovii*_2 | 11.147 | 10.37 | 98.73 | 95.03 | 93.02 |
| *S. krylovii*_3 | 11.139 | 10.31 | 98.72 | 95.16 | 92.59 |

Note: Raw Reads, measured in gigabases (Gb), represent the total volume of sequencing data obtained directly from the sequencer. These are unprocessed reads and include both high-quality and low-quality sequences. Clean Reads, also measured in gigabases (Gb), denote the portion of raw sequencing data that has undergone quality control and filtering to remove low-quality reads, adapters, and contaminants. These reads are considered suitable for subsequent analysis. Q20/Q30 is a measure of sequencing data quality and represents the percentage of bases with a Phred quality score of 20 or higher (Q20) and 30 or higher (Q30). The ratio represents the proportion of clean reads to the total raw reads, expressed as a percentage. It indicates the efficiency of data preprocessing and filtering, with a higher ratio indicating more successful data cleaning.

**Supplementary Tabel S2** Table of species BioProject and SRA Accession ID**.**

| Species | Sample ID | Accession ID | BioProject ID |
| --- | --- | --- | --- |
| *S. basiplumosa* | 1 | SAMN37333555 | PRJNA1014579 |
|  | 2 | SAMN37333556 |  |
|  | 3 | SAMN37333557 |  |
| *S. breviflora* | 1 | SAMN37341021 | PRJNA1014757 |
|  | 2 | SAMN37341022 |  |
|  | 3 | SAMN37341023 |  |
| *S. capillacea* | 1 | SAMN37333876 | PRJNA1014596 |
|  | 2 | SAMN37333877 |  |
|  | 3 | SAMN37333878 |  |
| *S. glareosa* | 1 | SAMN37334562 | PRJNA1014658 |
|  | 2 | SAMN37334563 |  |
|  | 3 | SAMN37334564 |  |
| *S. grandis* | 1 | SAMN37341044 | PRJNA1014760 |
|  | 2 | SAMN37341045 |  |
|  | 3 | SAMN37341046 |  |
| *S. krylovii* | 1 | SAMN37341977 | PRJNA1014801 |
|  | 2 | SAMN37341978 |  |
|  | 3 | SAMN37341979 |  |
| *S.* *tremula* | 1 | SAMN37342208 | PRJNA1014811 |
|  | 2 | SAMN37342209 |  |
|  | 3 | SAMN37342210 |  |
| *S. purpurea* | 1 | SAMN37334430 | PRJNA1014756 |
|  | 2 | SAMN37334431 |  |
|  | 3 | SAMN37334432 |  |
| *S. roborowskyi* | 1 | SAMN37341015 | PRJNA1014755 |
|  | 2 | SAMN37341016 |  |
|  | 3 | SAMN37341017 |  |
| *S. subsessiliflora* | 1 | SAMN37341018 | PRJNA1014613 |
|  | 2 | SAMN37341019 |  |
|  | 3 | SAMN37341020 |  |
| *S. gobica* | 1 | SAMN37341041 | PRJNA1014758 |
|  | 2 | SAMN37341042 |  |
|  | 3 | SAMN37341043 |  |
| *S. klemenzi* | 1 | SAMN37341974 | PRJNA1014800 |
|  | 2 | SAMN37341975 |  |
|  | 3 | SAMN37341976 |  |

Note: The “Sample ID” column provides unique identifiers for each biological replicate of the *Stipa* species. The “Accession ID” column contains the unique identifiers assigned to each sample in the Sequence Read Archive (SRA) database. These identifiers are used to access and retrieve the raw sequencing data associated with each sample. The “BioProject ID” column displays the BioProject ID associated with each Stipa species, it serves as a reference to the broader context of the study and can be used to access raw data and metadata.

**Supplementary Tabel S3** Table of *Stipa* voucher specimen numbers**.**

| Species | Voucher specimen numbers |
| --- | --- |
| *S. basiplumosa* | 2018-221 |
| *S. purpurea* | 2018-247 |
| *S. roborowskyi* | 2018-222 |
| *S. glareosa* | 2018-233 |
| *S. capillacea* | 2018-186 |
| *S. tremula* | 2019-050 |
| *S. subsessiliflora* | 2018-430 |
| *S. breviflora* | 2019-345 |
| *S. gobica* | 2019-346 |
| *S. klemenzi* | 2019-347 |
| *S. grandis* | 2019-401 |
| *S. krylovii* | 2019-402 |

Note: All specimens were stored in the herbarium of the College of Ecology and Environment, Inner Mongolia University

**Supplementary Figure S1.** BUSCO results of the assembled transcriptomes. Complete and Single-Copy (C and S): This means that the identified genes are complete and present as single copies in the sample genome. Complete and Duplicated (C and D): This indicates that the identified genes are complete, but there are multiple copies of them in the sample genome. Fragmented (F): This suggests that the identified genes are incomplete and may be fragmented into multiple pieces in the sample genome. Missing (M): This means that single-copy genes are absent or missing in the sample genome.

**Supplementary Figure S2.** Flowchart depicting the construction of phylogenetic trees for the *Stipa* genus. The flowchart illustrates the stepwise procedure for acquiring three distinct gene sets and the corresponding methodologies employed in formulating the systematic phylogenetic tree. The principal contrast within tree-building approaches lies in the selection of software, as well as the utilization of ModelFinder (Edge-linked mode) for partitioning and surveying evolutionary models. The Maximum Likelihood (ML) tree is generated through software RAxML and IQtree, while the Bayesian tree (BI tree) is derived using bayes. The terms "SCGs" and "One2" denote single-copy genes and one-to-one orthologous genes, respectively, serving as representatives.
