# Supplementary figures and images for: Elucidation of the evolutionary history of Stipa in China using comparative transcriptomic analysis
Source: Front Plant Sci. 2023 Nov 28;14:1275018. doi: 10.3389/fpls.2023.1275018 (PMC10751131; doi:10.3389/fpls.2023.1275018)

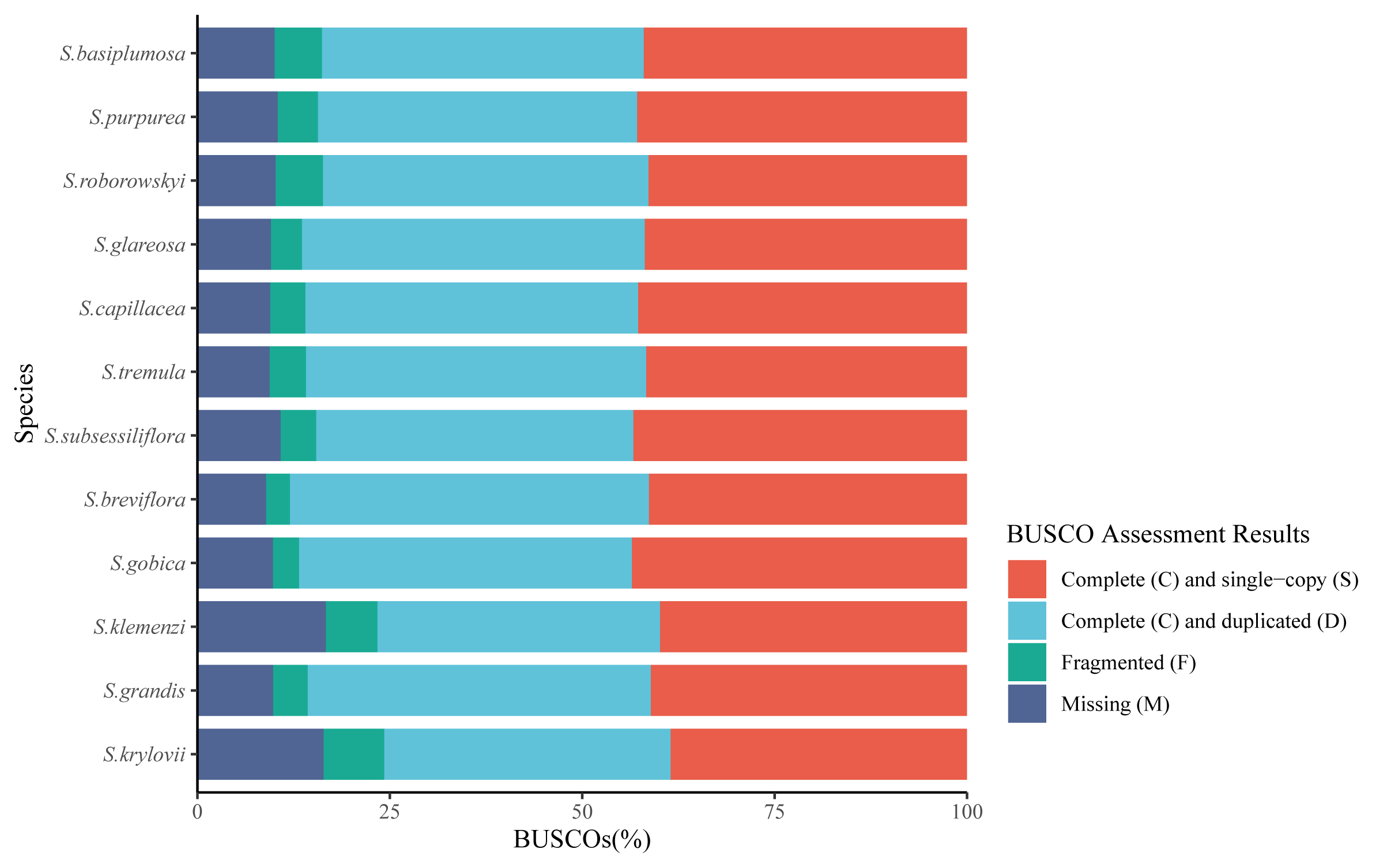

Supplement: Supplementary file 2 [file Image_1.tif]

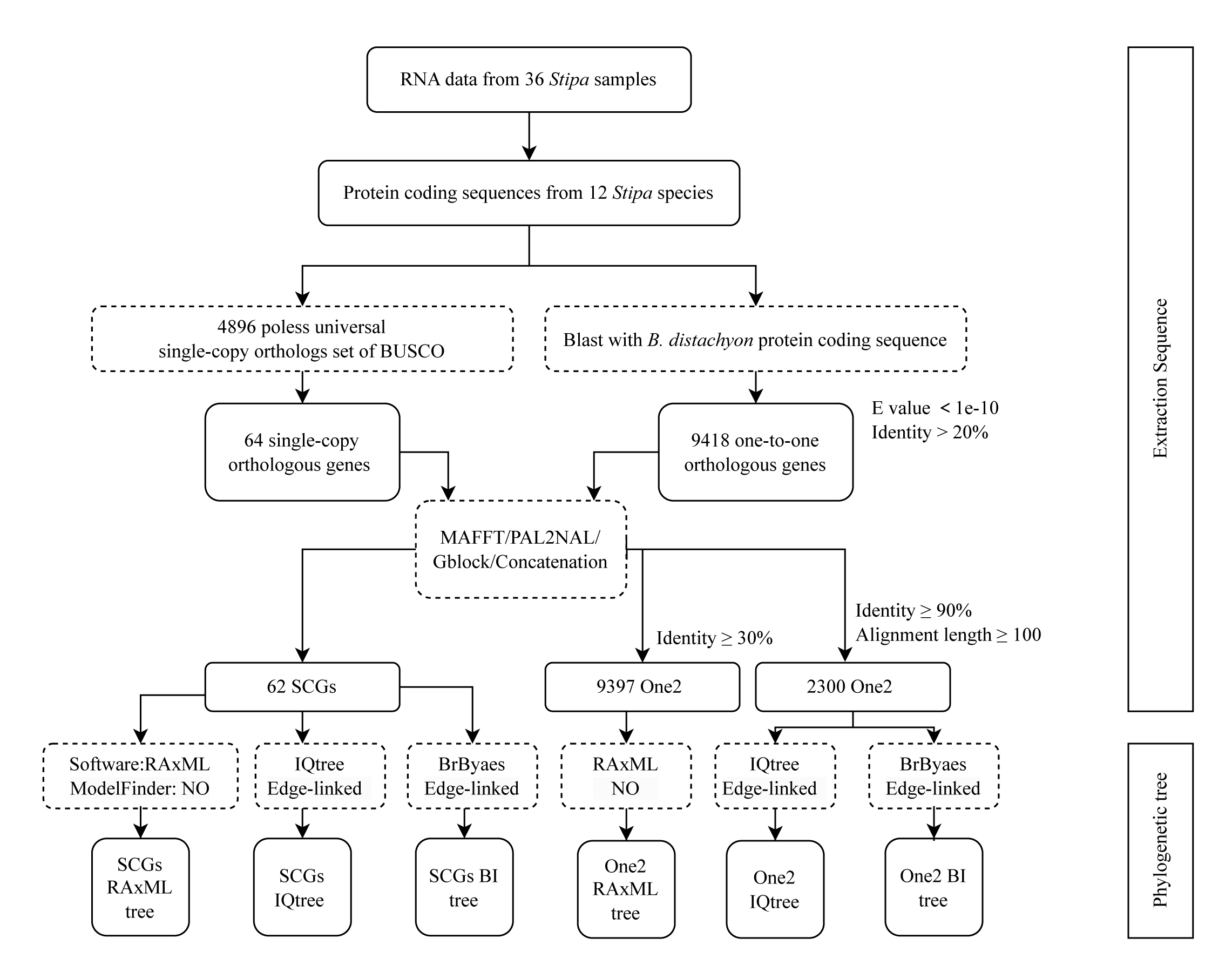

Supplement: Supplementary file 3 [file Image_2.tif]
